# Effective Stakeholder Engagement for Collation, Analysis and Expansion of Antimicrobial Resistance (AMR) Data: A CAPTURA Experience

**DOI:** 10.1093/cid/ciad585

**Published:** 2023-12-20

**Authors:** Nimesh Poudyal, Marianne Holm, Hea Sun Joh, Sanjay Gautam, Mohammad Julhas Sujan, Soo Young Kwon, Affan Sahikh, Alina Shaw, Partick Gallagher, Kristi Prifti, Alyssa Cho, Kyu-young Kevin Chi, Ahmed Taha Aboushady, William R MacWright, John Stelling, Florian Marks

**Affiliations:** International Vaccine Institute, Seoul, Republic of Korea; Research & Collaboration, Anka Analytica, Melbourne, Australia; International Vaccine Institute, Seoul, Republic of Korea; International Vaccine Institute, Seoul, Republic of Korea; International Vaccine Institute, Seoul, Republic of Korea; International Vaccine Institute, Seoul, Republic of Korea; Public Health Surveillance Group LLC, Princeton, USA; Public Health Surveillance Group LLC, Princeton, USA; Public Health Surveillance Group LLC, Princeton, USA; International Vaccine Institute, Seoul, Republic of Korea; International Vaccine Institute, Seoul, Republic of Korea; International Vaccine Institute, Seoul, Republic of Korea; International Vaccine Institute, Seoul, Republic of Korea; Brigham & Women's Hospital, Harvard Medical School, Boston, Massachusetts, USA; Public Health Surveillance Group LLC, Princeton, USA; Brigham & Women's Hospital, Harvard Medical School, Boston, Massachusetts, USA; International Vaccine Institute, Seoul, Republic of Korea; Cambridge Institute of Therapeutic Immunology and Infectious Disease, University of Cambridge School of Clinical Medicine, Cambridge, United Kingdom; Heidelberg Institute of Global Health, University of Heidelberg, Heidelberg, Germany; Madagascar Institute for Vaccine Research, University of Antananarivo, Antananarivo, Madagascar

**Keywords:** Stakeholder engagement, antimicrobial resistance, AMR, surveillance

## Abstract

**Background:**

An effective implementation of antimicrobial resistance (AMR) surveillance projects requires sustainable and multidisciplinary engagement with stakeholders from various backgrounds, interests and aims. The “Capturing Data on Antimicrobial resistance Patterns and Trends in Use in Regions of Asia” (CAPTURA) project, funded by the Fleming Fund, initially targeted 12 countries in South Asia (SA) and Southeast Asia (SEA) to “expand the volume of historical and current data on AMR and antimicrobial usage” and support local agencies through capacity building activities.

**Methods:**

In this article, we focus on early stakeholder engagement activities and present overall statistics on AMR data collated from 72 laboratories across seven countries. This included 2.3 million records of antimicrobial susceptibility testing (AST) data, which were curated, analyzed, and shared back to the facilities for informed decision making.

**Results:**

Approximately 98% of the data collated by CAPTURA originated from laboratories based in SA countries. Furthermore, country-wide data were analyzed to identify commonly reported pathogens in each country, followed by descriptions of AST practices and multidrug-resistant (MDR) pathogens. Overall, we found meager adherence to standard guidelines to perform and record AST results, and a significant number of MDR pathogens were reported.

**Conclusions:**

We conclude that close collaboration with the existing national mechanisms for identifying AMR data sources was crucial for the project's success. Although we show a vast retrospective dataset on AMR is available for data sharing in Asia, there remain critical gaps in data generation/management practice and analysis capacity for AMR data at most facilities.

## Teaser

This study highlights key stakeholder engagement activities for the “CAPTURA project implementation in target countries. In addition, we present the volume of antimicrobial resistance (AMR) data and briefly analyze information for common pathogens and their AMR patterns

Antimicrobial resistance (AMR) is a major global health security threat impeding sustainable development goals [1]. Most impacted are low- and middle-income countries (LMICs) in Sub-Saharan Africa (SSA) and South and Southeast Asia (SA/SEA) [2]. A robust AMR surveillance system is critical to understanding the burden and spread of drug-resistant microorganisms [3]. However, challenges remain to strengthen the capacity of laboratories to generate data, implement comprehensive surveillance strategies covering wider geographical networks [4], and conduct a sustainable collaboration with national and international partners [5].

Surveillance data generated through a concerted and collaborative effort informs the estimation and distribution of AMR. This is crucial to stimulate activities to prevent, control, and monitor AMR pattern changes and facilitate planning [6, 7]. Increasing the volume increases granularity to detect rapidly emerging local and global trends [8]. In LMICs, comprehensive knowledge of existing AMR data sources and their volume/quantity is lacking. The AMR containment efforts are guided by the National Action Plan for AMR-NAP, which aims to improve awareness of antibiotics usage, strengthen areas such as AMR surveillance, infection prevention and control, and promote investments in research and collaborative activities. The “Capturing Data on Antimicrobial resistance Patterns and Trends in Use in Regions of Asia” (CAPTURA) (Ref: Marianne paper, CLINID supplement) priority countries in SA and SEA developed their own AMR-NAP guiding AMR prevention and control activities. The NAPs’ envisioned establishment includes Multisectoral coordination/steering committees such as Antimicrobial Resistance Containment Multi-sectoral and Steering Committees (AMRCSCs) or similar structures that, with the support of national technical working groups (NTWGs) and national reference laboratories (NRLs), oversee AMR containment efforts in a majority of countries worldwide including LMICs. It is essential for donor-supported programs complementing AMR containment to engage closely with these existing bodies and ensure alignment with ongoing and planned activities for productive and sustainable outcomes. The CAPTURA grant aimed to expand the volume of previously unknown AMR data, assess the data quality and identify gaps in data management practice in the human health sector (REF: Marianne et al, CLINID Supplement).

Furthermore, the CAPTURA project collected 3 years of de-identified retrospective AMR, antimicrobial consumption (AMC), and antimicrobial use (AMU) data, assessed the quality of data sets and laboratories where data were collected and analyzed data within a central database. The raw and curated data, along with a comprehensive report containing the findings on trends and patterns of AMR, data and practice gaps, and recommendations to improve data quality, were shared back to data owners in each country. In this article, we broadly describe our stakeholder engagement approach and the outcomes of CAPTURA for identifying and expanding AMR data volume in participating countries. We focus on assignments at the AMRCSC level and present key findings of AMR data collected by CAPTURA for improved decision-making for future AMR surveillance and control activities.

## METHODS

The CAPTURA project strategy and coordination of activities were led centrally by the International Vaccine Institute (IVI) with specific technical input and support from the consortium partners. The Public Health Surveillance Group (PHSG) supported all in-country implementation activities, including developing and monitoring data collection tools. The IVI and PHSG each assigned a country lead to ensure continuity and dedicated attention throughout the project. In addition, an in-country team carried out the project's core activities, including travelling to project sites for scoping, landscape mapping, meetings with stakeholders/data owners, capacity building through numerous training sessions, and finally, data collection. Country-specific adaptations were made to the approach and methods based on contextual relevance. A detailed programmatic approach is explained elsewhere (Hea et al, CLINID Supplement and Marianne et al, CLINID Supplement).

The existing AMR-NAP was reviewed in each country to identify the existing structure and potential stakeholders, followed by exploring the feasibility and willingness to collaborate for activities within the scope of CAPTURA. The facilities with antimicrobial susceptibility test (AST) data were identified for collaboration through scoping and desktop review, as well as at the recommendation of country's NTWG as priority facilities in the country. The facilities included public laboratories fully supported by government providing routine health care services at subsidized rate, private facilities owned by individuals/conglomerates with no subsidies and private-public facilities partially supported by foundations providing routine health care services at reduced rates. To foster agreements and in-country approval of activities, including drafting a Collaboration Agreement (CA) and/or Data Transfer Agreement (DTA), a draft was shared with National Coordination Committees (NCCs) for feedback and final approval.

The DTA included provisions related to data ownership and conditions on data sharing between different facilities within the country or the public. The data owners (collaborators) determined the degree of data sharing governed by existing laws and acceptable norms in a particular country. Representatives from data owing entities and IVI were the signatories of the DTA in countries other than Bangladesh, where a tripartite agreement was reached with the engagement of the department hosting NCCs at the Ministry of Health and Family Welfare (MoHFW).

Ownership of the data was not transferred to any party, including IVI and its consortium partners. All data were deidentified before processing, and access to data was limited to authorized representatives from the CAPTURA consortium and regulatory authorities to permit project-related monitoring, audits and inspections. At the end of the CAPTURA project, data were exclusively used for curation and analysis. The raw and curated data sets, findings, and recommendations based on gaps identified in the data quality were sent back to the data owners for further use.

Data were collected/collated from laboratory logbooks or extracted from laboratory information systems (LISs) and shared with the CAPTURA team by each laboratory. Any technical assistance required during the process was provided in-person or virtually (due to travel restrictions imposed for containing COVID-19 pandemic) by the in-country/remotely-based CAPUTRA team. Deidentified data from each site were collated in a central database and uploaded into a secure cloud-based repository hosted and maintained by CAPTURA. Paper-based laboratory AST data were directly entered into the WHONET software (whonet.org), whereas electronic data were extracted in Microsoft Excel or converted into any other format compatible with BacLink software and exported into WHONET for analysis. The following factors were considered for the quality assessment of data sets received by CAPTURA: availability of priority data indicators for AMR, availability of clinical data, granularity, cleanliness, completeness, and volume. An automated system was built into the WHONET software to generate narrative reports highlighting the epidemiological and quality findings of the data.

Capacity-building activities were developed with collaborators, and participation was highly encouraged. Such activities (on-site and virtual) were mainly limited to on-the-job guidance and coaching (during the process of on-site data entry) on basic use of WHONET for AST data entry and analysis. Whenever possible and deemed necessary, the CAPTURA in-country team liaised with country AMR stakeholders, including AMR-NTWGs and/or Fleming Fund Country Grantee (FFCG), and shared project updates, including on-site data collection activities. Reports and relevant findings to inform policies and practices related to AMR and infection prevention and control were shared with the stakeholders. Furthermore, results publications and presentations supported individual researchers and country collaborators.

We used the WHONET data encryption feature to analyze AMR data to aggregate the datasets from different sites/countries. CAPTURA developed the “Quick Analysis of Antimicrobial Trends and Pattern” (QAAPT) software (https://qaapt.com), and Microsoft Excel was used to curate further the WHONET-generated output and populate final descriptive statistics. In addition, we used Highcharts, a JavaScript library (Highcharts.com), to generate the graphs. Moreover, the WHONET software was also used to develop the multidrug resistance (MDR) profiles.

## RESULTS

### Stakeholder Engagement

The CAPTURA's in-country scoping activities resulted in stakeholder identification and approval of project implementation, country-specific scope of the project, and project implementation approach. This resulted in the formulation of a country-specific Country Implementation Plan (CIP). Similarly, reviewing the AMR-NAP status and functional national structures in CAPTURA countries (Supplementary Table 1) remained vital to informing the project design/management process.

Out of 12 countries, the implementation plan presented by the CAPTURA project was approved by NCCs of 7 countries (Bangladesh, Bhutan, Indonesia, Laos, Nepal, Papua New Guinea, and Timor Leste). In Sri Lanka, provisional approval obtained for implementing the project activity at a national level had to be restricted to the private sector only. Furthermore, CIP was presented to the NCC, and country engagement was facilitated through the FFCGs in Pakistan and Vietnam. For various reasons, engagement in two countries (India and Myanmar) was limited to scoping activities. Through this engagement, CAPTURA was able to collect granular level (eg, bacterial culture and AST findings) from seven countries (Bangladesh, Bhutan, Laos, Nepal, Papua New Guinea, Sri Lanka, and Timor Leste) and aggregated antibiogram data from Pakistan. Compared to the number of facilities that signed the data transfer agreement (DTA), fewer facilities ended up sharing the AMR data in most countries (Table 1).

**Table 1.** Number of Facilities With Data Transfer Agreement (DTA) Sharing the Antimicrobial Resistance (AMR) Data

Country	Number of Facilities With DTA	Number of Facilities Sharing AMR Data
Bangladesh	37	34
Bhutan	1^a^	4
Laos	4	1
Nepal	29	28
Pakistan	3	3^b^
Papua New Guinea	5	1
Sri Lanka	5	3
Timor Leste	4	1

^a^Single DTA was signed with the Ministry of Health covering all the public health laboratories in the country.

^b^Only aggregated data were shared.

### Volume of Data

#### Overview of the Data Collected/Collated

**Figure 1.**
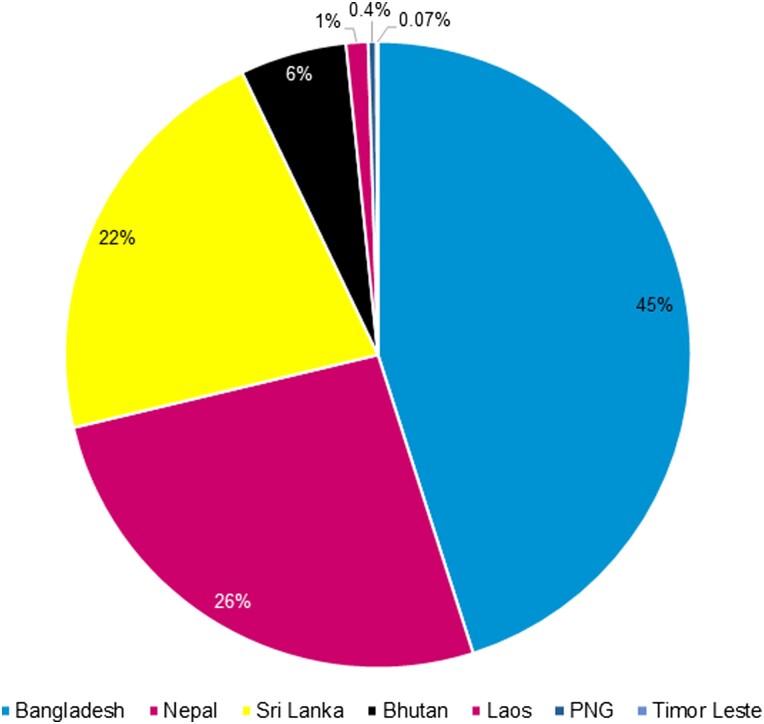
Distribution of data volume across countries. Abbreviation: PNG, Papua New Guinea.

During the country engagement period (May 2019 to March 2022), CAPTURA collected 2.37 million records of AST data from 72 sites (including healthcare-associated laboratories and stand-alone diagnostic laboratories) in seven countries. The majority (98%) of data originated from four countries (Bangladesh, Nepal, Sri Lanka, and Bhutan) in South Asia (Figure 1). Approximately 45% of total data were obtained from Bangladesh, followed by Nepal (26.1%), Sri Lanka (21.6%) and Bhutan (5.4%).

Approximately 58% of data were obtained from private facilities (Table 2). All of the facilities in Bhutan, Laos, Papua New Guinea, and Timor Leste were government-owned, whereas in Sri Lanka, data were exclusively obtained from private institutions. In Nepal, 3/28 facilities were under the public-private partnership policy.

**Table 2.** Number of Public, Private and Public-private Facilities Sharing Antimicrobial Susceptibility Test (AST) Data

Country	Public	Private	Public-Private	Total
Bangladesh	10	24	0	34
Bhutan	4	0	0	4
Laos	1	0	0	1
Nepal	10	15	3	28
Papua New Guinea	1	0	0	1
Sri Lanka	0	3	0	3
Timor Leste	1	0	0	1
Total	27	42	3	72

Furthermore, we analyzed the quality of AMR data based on their completeness in recording eight high-priority variables and reporting microbial findings at the species level (Table 3). Data completeness ranged from 75% in Nepal to 97% in Bangladesh. The most complete records shared from all countries were for the patient's age and sex, specimen type, specimen date, location, and microorganism type. A wide variation was observed for the variables, identification number (0% in Papua New Guinea to 100% in Bangladesh, Sri Lanka, and Timor Leste) and location type (29% in Bhutan to 99% in Bangladesh). Similarly, the characterisation of aerobic bacteria to species level varied between countries, ranging from at least 29% in Bangladesh to 98% in Papua New Guinea.

**Table 3.** Data Quality Metrices

	Bangladesh	Bhutan	Laos	Nepal	Papua New Guinea	Sri Lanka	Timor-Leste
Data completeness							
Age	100%	99%	99%	95%	84%	99%	99%
Microorganism type	100%	100%	99%	100%	100%	100%	99%
Identification number	100%	63%	13%	50%	0%	100%	100%
Sex	92%	99%	99%	98%	90%	95%	100%
Specimen type	97%	95%	89%	100%	100%	100%	100%
Specimen date	100%	100%	88%	91%	78%	100%	100%
Location	91%	100%	80%	26%	89%	100%	96%
Location type	99%	29%	72%	40%	89%	36%	60%
Overall	97%	86%	80%	75%	79%	91%	94%
Reporting microorganisms to species level	29%	77%	63%	74%	98%	47%	91%

#### Bacterial Culture

**Figure 2.**
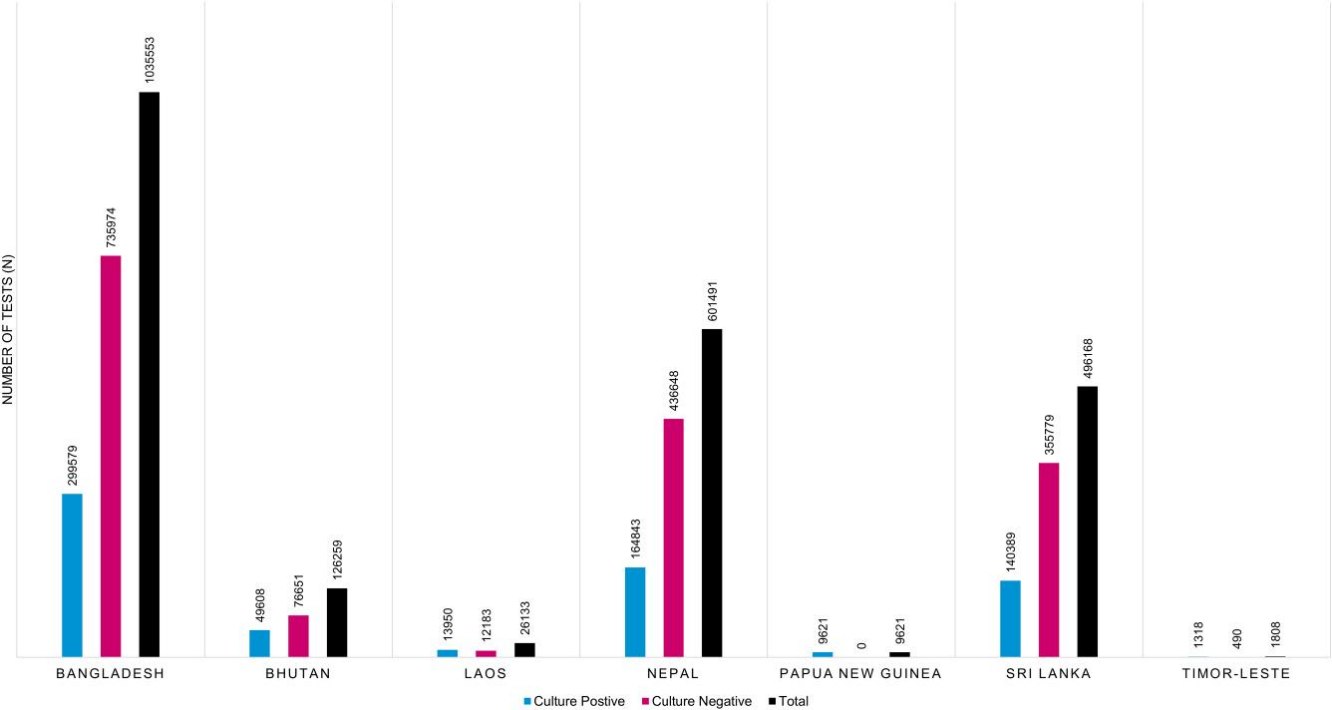
Total number of culture positive and culture negative test results reported in each country.

Results recorded for bacterial growth (positive) and no growth (negative) were extracted and analyzed for all countries (Figure 2). There were more negative compared to positive results in Bangladesh (72%), Bhutan (61%), Nepal (73%), and Sri Lanka (72%). However, Laos and Timor Leste reported higher positive cultures (53% and 72%, respectively). Data from Papua New Guinea warrants cautious interpretation as the facility only shared records on culture-positive cases. We further analyzed the distribution of bacterial culture data per country per year (Supplementary Figure 1, Supplementary Table 2), showing a relative decline in the total number of tests recorded in 2020 compared to earlier years, potentially related to the emergence of the COVID-19 pandemic.

**Figure 3.**
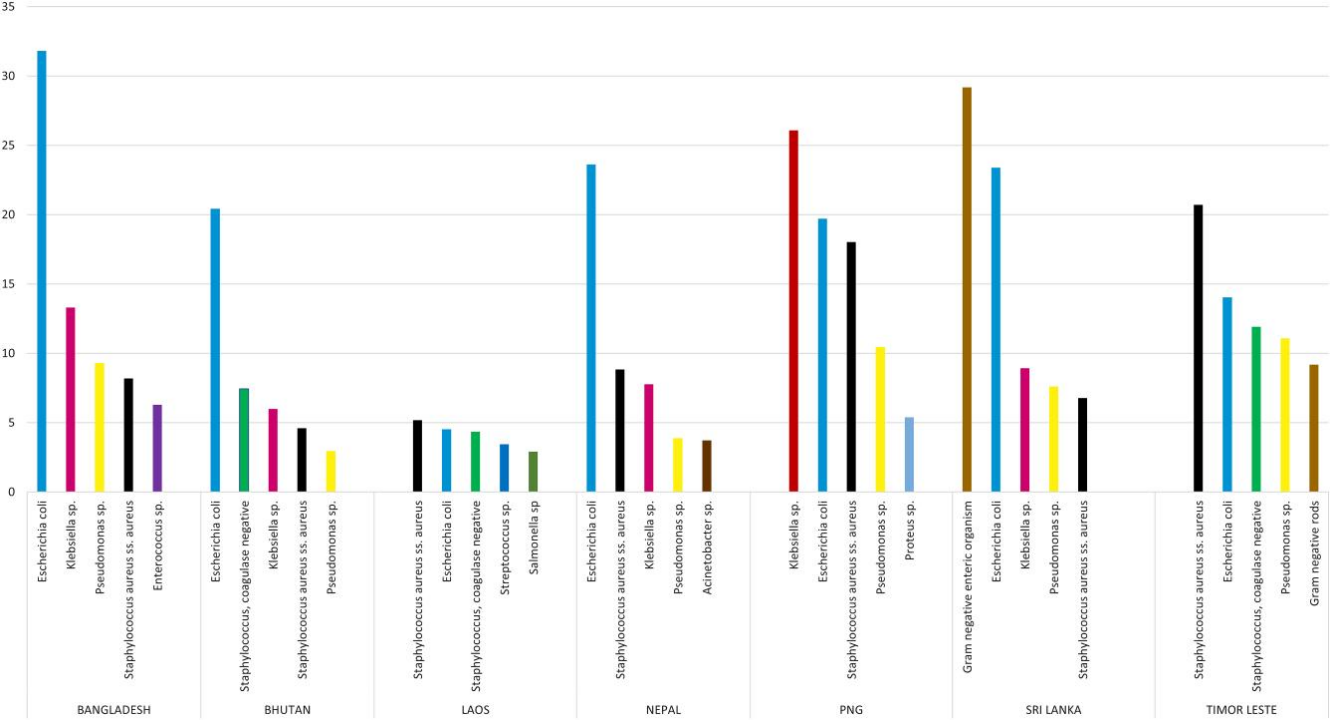
Proportion of five most commonly reported microorganisms in each country. Abbreviation: PNG, Papua New Guinea.

Next, the country-wide data were analyzed to identify commonly reported pathogens (Figure 3, Supplementary Table 3). Reporting of gram-negative bacilli (GNB) predominated in all datasets. *Escherichia coli* was reported most frequently in Bangladesh, Bhutan, and Nepal, whereas *Staphylococcus aureus* subspecies *aureus* prevailed in Timor Leste and Laos and *Klebsiella* sp. in Papua New Guinea. In Sri Lanka, the most commonly reported finding was a broad category reported as gram-negative enteric without further speciation. Overall, the five most frequent pathogens were nearly identical in most of the countries. It is also noted that urine was the most common specimen type, as evident in the data set received from SA countries, whereas, in SEA countries, it was not the case (Supplementary Figure 2).

#### Antibiotic Susceptibility Testing

**Figure 4.**
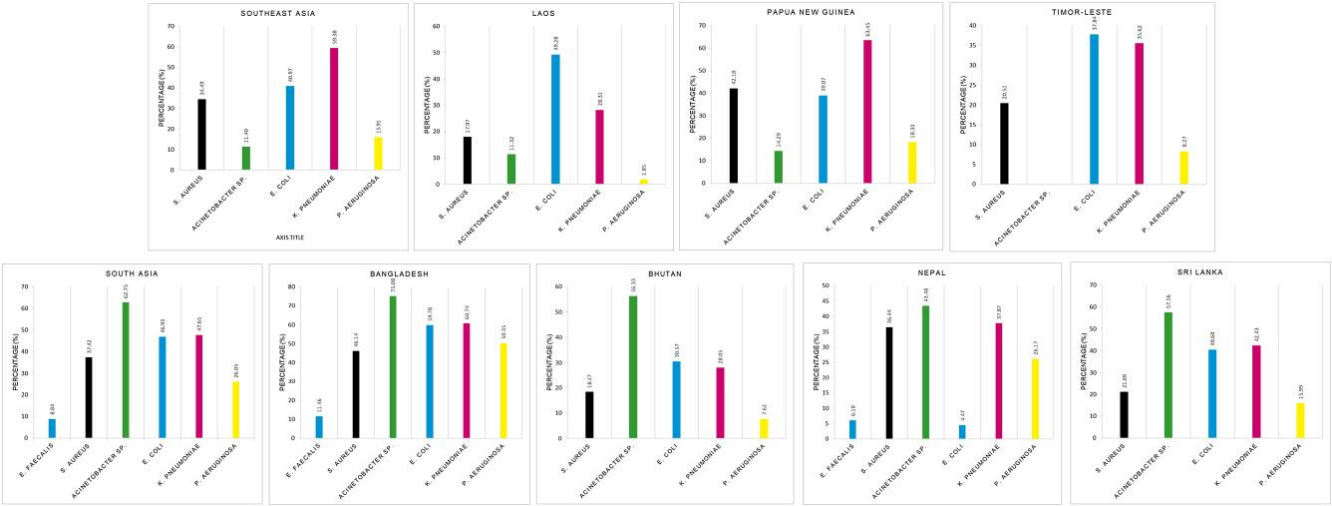
Five most commonly reported MDR microorganisms in each country. Abbreviations: E. FAECALIS, *Enterococcus faecalis*; E. COLI, *Escherichia coli*; K. PNEUMONIAE, *Klebsiella pneumoniae*; MDR, multidrug-resistant; P. AERUGINOA, *Pseudomonas aeruginosa*; S. AUREUS, *Staphylococcus aureus*.

Detailed analyses of resistance profiles on the isolated pathogens, including gram-positive and gram-negative antibiograms, were generated and shared with the laboratories. In terms of methods used for AST, disk diffusion was used in 71 out of 72 facilities across seven countries, and results were reported as resistant, intermediate, or sensitive to tested antibiotics. The five most commonly reported MDR pathogens included *Enterococcus faecalis*, *S. aureus*, *Acinetobacter* sp. *E. coli*, *Klebsiella pneumoniae*, and *Pseudomonas aeruginosa* (Figure 4, Supplementary Table 4). Only one laboratory shared AST records with zone diameter with CAPTURA, and none of the data sets had data related to routine internal quality control (IQC) tests performed at those laboratories. Although most laboratories responded during a survey of conducting regular IQC testing, the inability to review and analyze the IQC tests was one of the main reasons for not being able to assess the quality of retrospective data collected by CAPTURA. Furthermore, using CAPTURA-developed analytical functionality in the WHONET application, the CAPTURA team generated an “Epidemiological, Test Practice and Data Quality” report for each data set obtained. A similar report was also prepared after aggregating records from the data set. The subsequent section provides additional information on the quality and epidemiology pattern based on the CAPTURA-supported WHONET-generated report. This functionality is now embedded in the WHONET program and can be run on any dataset to generate these reports in future.

The AST data shared by each country was analyzed for the WHO list of global priority pathogens under three priority tiers to their antibiotic resistance profile: critical, high and medium (Table 4). Three critical pathogens, carbapenem-resistant *Acinetobacter* spp, ceftriaxone-resistant *E. coli* and meropenem-resistant *E. coli,* were commonly recorded across all countries. Carbapenem-resistant *P. aeruginosa* and cefotaxime-resistant *E. coli* were reported from three and five countries, respectively. Similarly, high-priority *Salmonella* spp. (fluroquinolone resistant (ciprofloxacin)) was prevalent in all countries, whereas clarithromycin-resistant *Helicobacter pylori* and fluoroquinolone-resistant (ciprofloxacin) *Campylobacter* spp. remain largely unrecorded in all countries in the available data set. Furthermore, the data showed the varied prevalence of the medium-priority three pathogens in all countries except Timor-Leste.

**Table 4.** The Highest and Lowest Reported Prevalence of MDR-bacteria (WHO Priority List and Indicators for UN SDG) in CAPTURA Countries*

Multidrug Resistant Bacteria	Highest (%)	Lowest (%)	Data Unavailable/insufficient (n, Countries)	WHO Priority
WHO priority list of antibiotic-resistant bacteria				
*Acinetobacter* spp. (Carbapenem-resistant)	56	17	NA	Critical
*Pseudomonas aeruginosa* (Carbapenem-resistant)	65	16	4	Critical
*Escherichia coli* (Cefotaxime-resistant)	64	33	2	Critical
*Escherichia coli* (Ceftriaxone-resistant)	61	34	NA	Critical
*Escherichia coli* (Meropenem-resistant)	10	1	NA	Critical
*Enterococcus faecium* (Vancomycin-resistant)	50	0	2	High
*Staphylococcus aureus* (Methicillin-resistant)	51	18	1	High
*Staphylococcus aureus* (Vancomycin-resistant)	5	0	2	High
*Staphylococcus aureus* (Vancomycin-intermediate)	9	0	2	High
*Helicobacter pylori* (Clarithromycin-resistant)	NA	NA	7	High
*Campylobacter* spp. (Fluoroquinolone-resistant)	75	NA	6	High
*Salmonella* spp. (Fluoroquinolone-resistant (Ciprofloxacin))	37	4	NA	High
*Neisseria gonorrhoeae* (Third generation cephalosporin-resistant)	14	0	2	High
*Neisseria gonorrhoeae* (Fluoroquinolone-resistant)	62	22	2	High
*Streptococcus pneumoniae* (Penicillin non-susceptible)	40	0	1	Medium
*Haemophilus influenzae* (Ampicillin-resistant)	37	11	2	Medium
*Shigella* spp. (Fluoroquinolone-resistant)	79	5	1	Medium
Indicators for UN SDG				
MRSA in blood (Oxacillin)	47	40	5	N/A
MRSA in blood (Cefotaxin)	48	8	2	N/A
Third-generation cephalosporin-resistance *Escherichia coli* in blood	66	37	2	N/A

Abbreviations: CAPTURA, Capturing Data on Antimicrobial resistance Patterns and Trends in Use in Regions of Asia; MRSA, methicillin-resistant *Staphylococcus aureus*; N/A, not applicable; UN SDG, United Nations Sustainable Developments Goals; WHO, World Health Organization.

^a^Percentage is calculated from shared dataset, the size of which varied between countries.

The percentage of bloodstream infection due to methicillin-resistant *S. aureus* (MRSA) and *E. coli* resistant to third-generation cephalosporin (eg, ESBL- *E. col*i) was further evaluated as an indicator for the United Nations Sustainable Developments Goals (UN-SDG). The records showed the presence of Oxacillin-resistant MRSA in Bangladesh and Nepal and Cefoxitin-resistant MRSA strains in all countries except Sri Lanka and Timor-Leste. For ESBL- *E. coli,* no results were found for Bangladesh, and insufficient data were available for Timor Leste (Table 4).

In addition, AMR capacity-building activities were conducted in laboratories in Bangladesh (n = 49), Bhutan (n = 4), Laos (n = 13), Nepal (n = 21), Papua New Guinea (n = 1), Sri Lanka (n = 4), and Timor Leste (n = 5). Details on capacity-building activities and follow-up assessments is due to be published elsewhere.

## DISCUSSION

Implementing the CAPTURA project in different countries required a collaborative effort with multiple stakeholders to conduct activities such as collating the AMR data, fostering capacity-building activities and advocating for improved AMR surveillance programs. This study presents early stakeholder engagement activities that led to the CAPTURA project's launch in respective countries. Marriane et al (CLINID supplement) and Hae et al (CLINID supplement) present in detail the collaborative approach to collect, store, transfer, and analyze the data and conduct capacity-building programs.

Successful employment of multi-country projects such as the CAPTURA is challenged by technical, organizational, and sociological elements, mainly due to the engagement of stakeholders from multidisciplinary backgrounds and interests [9]. Furthermore, inadvertent situations such as the COVID-19 pandemic pose unprecedented context. Therefore, each country's engagement methods and approaches were contextually adapted wherever needed. Throughout the project, unremitting communications between the CAPTURA core team at IVI, CAPTURA's in-country team and other relevant in-country entities (national stakeholders and multinational/international agencies) actively working in the field of AMR in all countries were ensured. To receive a comprehensive update on AMR activities in each country, the Library of AMR-NAPs approved by the World Health Organization (WHO) [10] was referenced.

CAPTURA's successful implementation can be attributed to early stakeholder engagement and a locally embedded collaborative approach. Nearly all the activities developed under CAPTURA CIPs were aimed at supporting the country's NAP activities. Furthermore, identification, extraction, analysis, and interpretation of data, along with an assessment of its quality, was one of the critical components of the project that attracted stakeholder's interest as this posed an opportunity to understand the country's existing capacity in data generation, which could be considered as a baseline and potential for establishment and/or expansion of AMR surveillance network in each country. Similarly, a collaborative approach for capacity building focused on digitalising retrospective AMR data and hands-on training for prospective data management, data extraction, analysis, and reporting/dissemination further led to the project's acceptance and success regionally.

We show AMR data stored in local repositories in participating countries. Due to differing levels of engagement and data types, the information generated and presented by CAPTURA needs cautious interpretation as this cannot be generalized for a country as a whole, nor is it relatively interpreted between the countries. For example, CAPTURA collected entire retrospective data from four laboratories in Bhutan. As those four laboratories were the only ones generating AST data in the human health sector at the time of collection, the findings of the data can be generalized as national data. Although we were able to collate data from only one facility each in Timor Leste and Papua New Guinea, this can also be interpreted as national findings because these were the only facilities with the capacity to produce reliable and regular AST data in the country at the time of CAPTURA implementation. Conversely, CAPTURA collected data from 28 and 34 major healthcare providers in Nepal and Bangladesh, but these numbers only represent a fraction of facilities generating AST data in these countries.

Similarly, we assessed the data quality based on their completeness. Data completeness ranged from 75% in Nepal to 97% in Bangladesh. Acharya and co-workers [11] from Nepal reported 88%–100% complete data from five surveillance sites in Province 3 and highlighted the need for “improvement in completeness, consistency, and timeliness’ of AMR surveillance data. Although inter-site variation in data completeness is reported in various settings [11, 12], such difference in the current study is explained by the ranging capacity of laboratories in each country. In any case, the provision to record and report complete data should remain a critical component in AMR surveillance as this generates reliable results to analyse and inform policy [13]. The information in this study is potentially impacted by biases as the CAPTURA approach for selecting sites for data collection was based on convenience and willingness to collaborate, thus lacking a systematic representative approach to generalize the findings at the national level in these countries. However, overall, we observed the willingness and capacity of participating laboratories toward sharing their data at the national and potentially regional level. Therefore, continuous advocacy engagement with sites and national stakeholders may be possible in SA for establishing regional surveillance.

A majority of data obtained by the CAPTURA was sourced from private facilities that were primarily not participating in existing AMR surveillance programs in the country. The data from all countries shows frequent reporting of common pathogens such as *E. coli, P. aeruginosa,* and *Staphylococcus* species. This potentially indicates the predominance of such microorganisms in the community or due to relative technical ease in laboratory procedure compared to pathogens such as *Streptococcus* species, *Neisseria* species, and *Haemophilus* species requiring additional resources for growth and identification. Next, the capacity to report bacteria to species level was used as a proxy to evaluate the performance of the participating facility. Data were analyzed for aerobic bacteria except for *S. aureus* and *E. coli,* as these bacteria are commonly differentiated to species level. Although 29% of total microorganisms reported in Bangladesh were characterized by species level, this remained at 98% in Papua New Guinea.

Similarly, data shared from individual countries show a considerable burden of MDR microorganisms, predominantly those included in the WHO priority pathogens list of antibiotic-resistant bacteria [14] and UN-SDG. The data further depict variation in countries reporting the prevalence of these pathogens. For example, clarithromycin-resistant *Helicobacter pylori,* which is a microaerophilic organism, was recorded in none of the seven countries. This warrants an urgent priority for laboratory capacity building for constant surveillance to prevent the local spread and outbreak of pathogens with limited antibiotic options.

One key observation on the data set shared by collaborating facilities was missing records for internal quality control (IQC) test findings. Although most facilities responded that they performed IQC (Hea et al, CLINID Supplement), the inability to share or maintain these data prevented future retrospective quality checks. Furthermore, very few facilities were recording zone of inhibition in their database; this not only leads to the inability to assess the data retrospectively but also prevents reassessment of resistance pattern should there be any change in breakpoints and the zone of diameter for resistance, susceptible, or intermediate for any particular antibiotic.

CAPTURA's findings demonstrate the availability of bacteriological culture and AST records in at least 72 facilities across CAPTURA priority countries in SA and SEA. These numbers are incomplete as CAPTURA identified additional facilities in the region as potential data generation sites (Sanju et al, CLINID supplement) as demonstrated in the paper by Hae et al (CLINID supplement). Each country needs to maintain an updated list of all facilities generating AMR data to understand the country's capacity and prepare policies based on existing strengths. Similarly, a significant gap in routine AMR data management practice and IQC has been observed in almost all the countries in the region. Multiple data management systems are being used in the same country, resulting in a non-uniform structure of generated data. Data curation was the most cumbersome and time-consuming process experienced by the CAPTURA team, and the first complete analysis post-data collection required nearly 10 months (June 2022 to March 2023). Going forward, countries should consider defining a minimum set of essential variables to standardize the data output at the country level, thus minimizing the requirement for extensive data curation. Similarly, the focus should not only be concentrated on increasing the quantity of data, but equal efforts should also be made to improve data quality by implementing robust quality management practices in each facility, thus upgrading the quality of microbiology diagnostic services.

## Supplementary Data

Supplementary materials are available at *Clinical Infectious Diseases* online. Consisting of data provided by the authors to benefit the reader, the posted materials are not copyedited and are the sole responsibility of the authors, so questions or comments should be addressed to the corresponding author.

## Supplementary Material

ciad585_Supplementary_Data

## Notes

***Disclaimer.*** The views expressed here are those of the authors and not necessarily those of the UK Department of Health and Social Care or its management agent, Mott MacDonald; and, International Vaccine Institute.

***Acknowledgments.*** The authors would like to thank all of our collaborators who provided valuable support for successful implementation of the CAPTURA project, shared their data for curation and analysis, and local CAPTURA team members who have made invaluable contributions to the conduct of site visits, trainings, and data collection activities for the project.

***Supplement sponsorship.*** This article appears as part of the supplement “Capturing Data on Antimicrobial Resistance Patterns and Trends in Use in Regions of Asia (CAPTURA),” funded by the Department of Health and Social Care's Fleming Fund using UK aid.

***Financial support.*** This work was supported by the Fleming Fund regional grants (grant numbers FF10-135 and FF11-139). The Fleming Fund is a UK aid investment to support surveillance of antimicrobial resistance in low- and middle-income countries in Africa and Asia. The regional grants program is managed by Mott MacDonald Ltd, the Fleming Fund Grants Management Agent.
