# Supplementary material for: Effective Stakeholder Engagement for Collation, Analysis and Expansion of Antimicrobial Resistance (AMR) Data: A CAPTURA Experience
Source: Clin Infect Dis. 2023 Dec 20;77(Suppl 7):S519–27. doi: 10.1093/cid/ciad585 (PMC10732561; doi:10.1093/cid/ciad585)
Supplement: ciad585_Supplementary_Data [file ciad585_supplementary_data.zip › Nimesh et al_Supplementary Information.docx]

**Supplementary Table 1.** Current AMR-NAP status and functional governance structure in CAPTURA countries

| **Region** | **Country** | **AMR-NAP** | **National Reference Centre** | **Technical Committees overseeing HH sector** | **Other key AMR stakeholders** |
| --- | --- | --- | --- | --- | --- |
| SA | Bangladesh (15, 16) | - Approved NAP 2017-22 | - National Steering Committee (NSC) - Inter-ministerial level highest executive body | - National Technical Committee - Highest multisectoral, multidisciplinary executive technical body - Headed by Director level National AMR focal at DGHS, MoHFW | NGOs, INGOs, International agencies (WHO/FAO), The Fleming Fund-UKaid, USAID |
|  |  | - Draft of National AMR surveillance strategy 2020-25 | - National Coordination Centre (NCC) led by Director (Disease Control) at DGHS for AMR - National Regulatory Authority (NRA) at DGDA, MoHFW | - Sectoral Coordination Center for HH led by sectoral coordinator at IEDCR for AMR - DGDA for AMC/U |  |
|  | Bhutan (17) | - Approved NAP 2018-22 | - Drug Technical Advisory Committee (DTAC) as NSC | - AMR program at MoH as national focal agency and national AMR focal in AMR program | The Fleming Fund-UKaid |
|  | India (18) | - Approved NAP 2017-21 | - Intersectoral Coordination Committee on AMR (ICC-AMR); Technical Advisory Group (TAG-AMR) providing technical advice to ICC-AMR - Oversee and coordinate policy decisions | - National Centre for Disease Control (NCDC) designated as NCC with Core Working Group (CWG-AMR) providing technical and operational inputs | International agencies (WHO, FAO, OIE, UNEP), The Fleming Fund-UKaid, |
|  | Nepal(19) | - Draft National Antimicrobial Resistance Containment Action Plan 2016 (endorsement to be done by Nepal’s cabinet) | - AMR Steering Committee at MoH for coordination with all stakeholders | - National Antimicrobial Surveillance Centre at National Public Health Laboratory | NGOs, International agencies (WHO, FAO), USAID, The Fleming Fund_UKaid, |
|  |  | - Draft NAP 2021-26 (Finalized, to be endorsed by Nepal’s cabinet) | - High-level Multisectoral Steering Committee – AMR (AMRMSC) chaired by Secretary of Health at MoH | - National Technical Working Committee (NTWC) in One Health approach chaired by chief of QSRD at MoHP and TWC for each strategic priority |  |
|  | Pakistan (20) | - Approved NAP 2017 | - National Institute of Health (NIH) Pakistan designated as national focal point for AMR | - Focal at NIH | International agencies (WHO), US-CDC, The Fleming Fund-UKaid |
|  | Sri Lanka (21) | - Approved National Strategic Plan (NSP) for Combating AMR 2017-22 | - National Advisory Committee on AMR (NAC-AMR) - multisectoral representation - National Action Plan Implementation Strengthening Team (NAPIST) – multisectoral team for implementation of activities identified in NSP | - Deputy Director General (Laboratory Services) as National focal point for AMR | International agencies (WHO, FAO) |
| SEA | Indonesia (22, 23) | - Approved NAP 2017-19 | - High Level Inter-Ministerial Steering Committee (IMSC) coordinated by Minister of Human Development and Culture - National Antimicrobial Resistance Coordination Committee (NARCC) | - ARCCs of MoH | International agencies (WHO, FAO), USAID, The Fleming Fund-UKaid, Australian Government |
|  |  | - Approved NAP 2020-24 | - AMR control task force consisting steering committee coordinated by minister of Human Development and Cultural Affairs - Multisectoral inter-ministerial committee for providing direction to executive committee, support on policy on AMR control and monitoring and evaluation | - Multi sectoral executive committee under the chairmanship of DGHS, Ministry of Health |  |
|  | Laos (24) | - Approved NAP 2019-23 | - Antimicrobial Resistance Surveillance and Control Committee (ASCC) led by Director General of Department of Communicable Disease Control | - ASCC sub-committee | Foundation Merieux, Institute Pasteur, LOMWRU, International agencies (WHO, FAO, OIE), The Fleming Fund-UKaid |
|  | Myanmar (25) | - Draft NAP 2017-22 | - National Multisectoral Steering Committee (NMSC) under leadership of MoHS | - National AMR Coordinating Centre (NACC) with TWGs for individual strategic objectives as implementing agency of NAP | International agencies (WHO, FAO) |
|  | Papua New Guinea (26) | - Approved NAP 2019-23 | - National AMR Steering Committee as highest level of governance reporting to Minister of Health; rotating chair between sector | - Technical Working Group at National Department of Health (NDoH) overseeing implementation of national AMR response | International agencies (WHO, FAO, OIE), Burnet Institute, Medicines Sans Frontier (MSF), The Fleming Fund-UKaid |
|  | Timor Leste (27) | - Approved NAP 2017-20 | - High level National Multisectoral Committee (NMC) – a group of political member, policy makers and program managers as implementation agency for NAP | - Multi-sectoral Technical Working Groups (TWGs) addressing strategic objectives through specialized Task Forces | International agencies (WHO, FAO. OIE), |
|  | Vietnam (28,29) | - Approved NAP 2013-20 | - Steering committee led by Minister of Health – multisectoral representation | - Sub-committees for with Department of Examination and treatment administration under MoH for establishment of surveillance | WHO, UN forestry fund, GARP-Vietnam, UNAIDS, WB, SIDA, The Fleming Fund-UKaid, Oxford University Clinical Research Unit, US-CDC, French Agricultural Research Centre for International Development (CIRAD) |

**Supplementary Table 2**. Yearly distribution of culture positive and culture negative reports in each country.

| **Country** | **Year** | **Culture Positive (n)** | **Culture Negative (n)** |
| --- | --- | --- | --- |
| Bangladesh | | | |
|  | 2016 | 2839 (40%) | 4269 (60%) |
|  | 2017 | 61857 (31%) | 140760 (69%) |
|  | 2018 | 92665 (30%) | 215390 (70%) |
|  | 2019 | 104375 (27%) | 281007 (73%) |
|  | 2020 | 37833 (29%) | 91939 (71%) |
|  | 2021 | 10 (0.4%) | 2609 (99.6%) |
| Bhutan | | | |
|  | 2017 | 16416 (37%) | 27514 (63%) |
|  | 2018 | 19937 (39%) | 31705 (61%) |
|  | 2019 | 13255 (43%) | 17432 (57%) |
| Laos | | | |
|  | 2018 | 1849 (89%) | 222 (11%) |
|  | 2019 | 12101 (50%) | 11961 (50%) |
| Nepal | | | |
|  | 2017 | 32467 (26%) | 92543 (74%) |
|  | 2018 | 48845 (26%) | 138437 (74%) |
|  | 2019 | 74217 (29%) | 183357 (74%) |
|  | 2020 | 9314 (29%) | 22311 (71%) |
| PNG | | | |
|  | 2016 | 469 (100%) | 0.00 (0%) |
|  | 2017 | 2594 (100%) | 0.00 (0%) |
|  | 2018 | 3219 (100%) | 0.00 (0%) |
|  | 2019 | 3339 (100%) | 0.00 (0%) |
| Sri Lanka | | | |
|  | 2018 | 49300 (29%) | 121751 (71%) |
|  | 2019 | 51356 (29%) | 128393 (71%) |
|  | 2020 | 39733 (27%) | 105635 (73%) |
| Timor-Leste | | | |
|  | 2016 | 248 (100%) | 0.00 (0%) |
|  | 2017 | 266(100%) | 0.00 (0%) |
|  | 2018 | 228(100%) | 0.00 (0%) |
|  | 2019 | 345 (41%) | 490 (59%) |
|  | 2020 | 231(100%) | 0.00 (0%) |

**Supplementary Table 3.** Proportion of commonly reported microorganisms among all positive cultures.

| Country | Microorganisms | Common pathogens (%) |
| --- | --- | --- |
| BANGLADESH | | |
|  | E. coli | 31.83 |
|  | Klebsiella sp. | 13.3 |
|  | Pseudomonas sp. | 9.3 |
|  | S. aureus ss. aureus | 8.19 |
|  | Enterococcus sp. | 6.3 |
| BHUTAN | | |
|  | E. coli | 20.43 |
|  | Coagulase Negative Staphylococcus | 7.44 |
|  | Klebsiella sp. | 5.99 |
|  | S. aureus ss. aureus | 4.61 |
|  | Pseudomonas sp. | 2.96 |
|  |  |  |
| LAOS | | |
|  | S. aureus ss. aureus | 5.19 |
|  | E. coli | 4.53 |
|  | Coagulase Negative Staphylococcus | 4.34 |
|  | Streptococcus sp. | 3.44 |
|  | Salmonella sp | 2.92 |
| NEPAL | | |
|  | E. coli | 23.63 |
|  | Coagulase Negative Staphylococcus | 8.85 |
|  | Klebsiella sp. | 7.78 |
|  | Pseudomonas sp. | 3.88 |
|  | Acinetobacter sp. | 3.73 |
| PNG | | |
|  | Klebsiella sp. | 26.09 |
|  | E. coli | 19.71 |
|  | Coagulase Negative Staphylococcus | 18.02 |
|  | Pseudomonas sp. | 10.45 |
|  | Proteus sp. | 5.4 |
| SRI LANKA | | |
|  | Gram Negative Enteric | 29.18 |
|  | E. coli | 23.4 |
|  | Klebsiella sp. | 8.94 |
|  | Pseudomonas sp. | 7.62 |
|  | S. aureus ss. aureus | 6.79 |
| TIMOR LESTE | | |
|  | S. aureus ss. aureus | 20.71 |
|  | E. coli | 14.04 |
|  | Coagulase Negative Staphylococcus | 11.91 |
|  | Pseudomonas sp. | 11.08 |
|  | Gram Negative rods | 9.18 |

**Supplementary Table 4.** Five most commonly reported multi-drug resistant microorganisms

| **Microorganisms** | **Bhutan** | **Bangladesh** | **Nepal** | **Sri Lanka** | **Laos** | **TL** | **PNG** |
| --- | --- | --- | --- | --- | --- | --- | --- |
| *E. faecalis* | - | 581 (11%) | 36 (6%) | - | - | - | - |
| *S. aureus* | 422 (18%) | 11,377 (46%) | 5913 (36%) | 2,008 (21%) | 123 (19%) | 12 (15%) | 707 (42%) |
| *Acinetobacter* sp. | 339 (56%) | 7,497 (76%) | 3435 (50%) | 1,124 (57%) | 18 (13%) | 5 (50%) | 74 (21%) |
| *E. coli* | 2,604 (26%) | 57,237 (60%) | 11933 (27%) | 13,339 (41%) | 279 (49%) | 23 (43%) | 805 (45%) |
| *K. pneumoniae* | 605 (23%) | 8,116 (61%) | 4052 (38%) | 1,623 (42%) | 56 (21%) | 8 (47%) | 1483 (64%) |
| *P. aeruginosa* | 58 (8%) | 1,945 (50%) | 1361 (26%) | 1,278 (16%) | 2 (2%) | - | 166 (19%) |
