# Supplementary figures and images for: Effective Stakeholder Engagement for Collation, Analysis and Expansion of Antimicrobial Resistance (AMR) Data: A CAPTURA Experience
Source: Clin Infect Dis. 2023 Dec 20;77(Suppl 7):S519–27. doi: 10.1093/cid/ciad585 (PMC10732561; doi:10.1093/cid/ciad585)

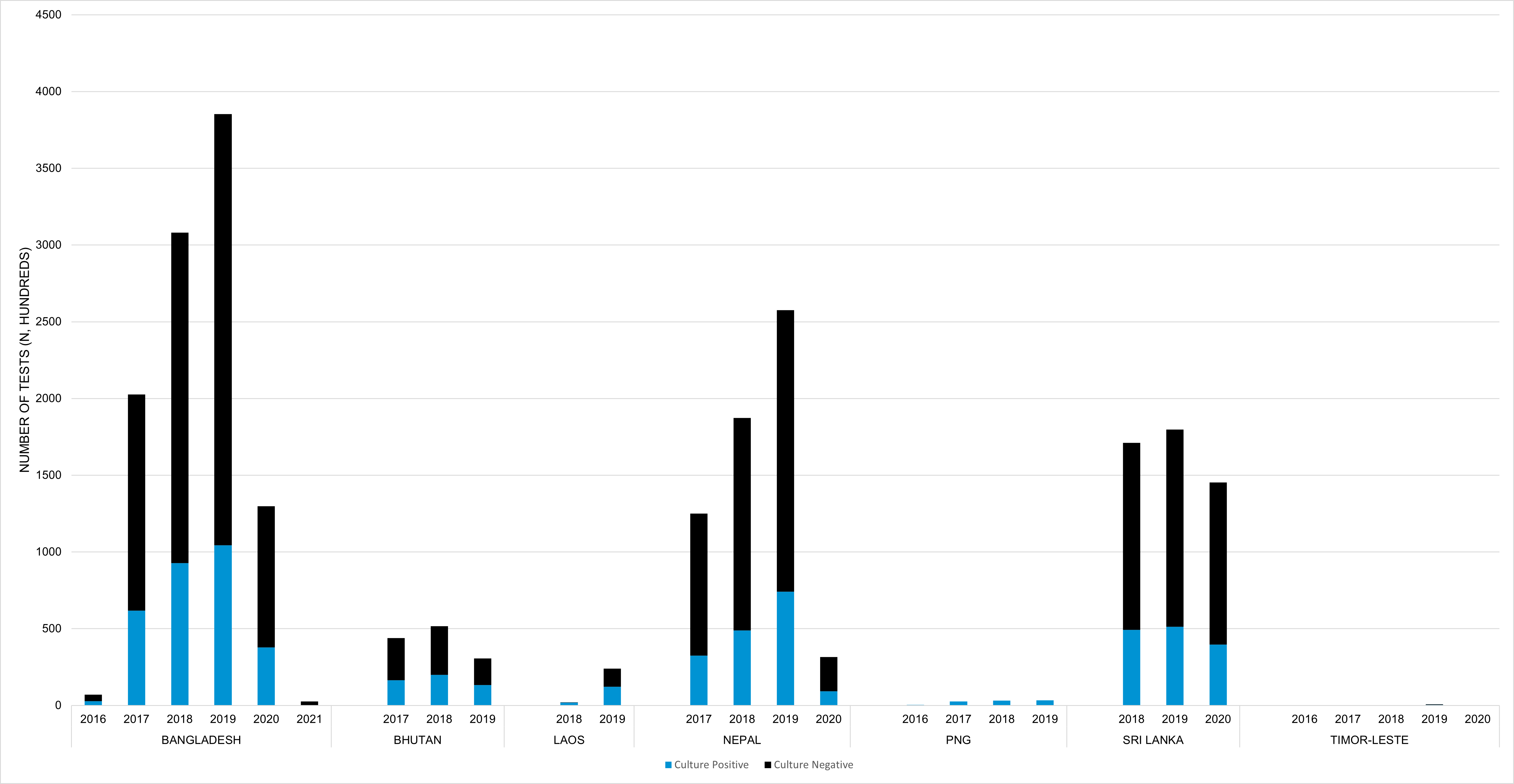

Supplement: ciad585_Supplementary_Data [file ciad585_supplementary_data.zip › Supplementary Figure 1.tif]

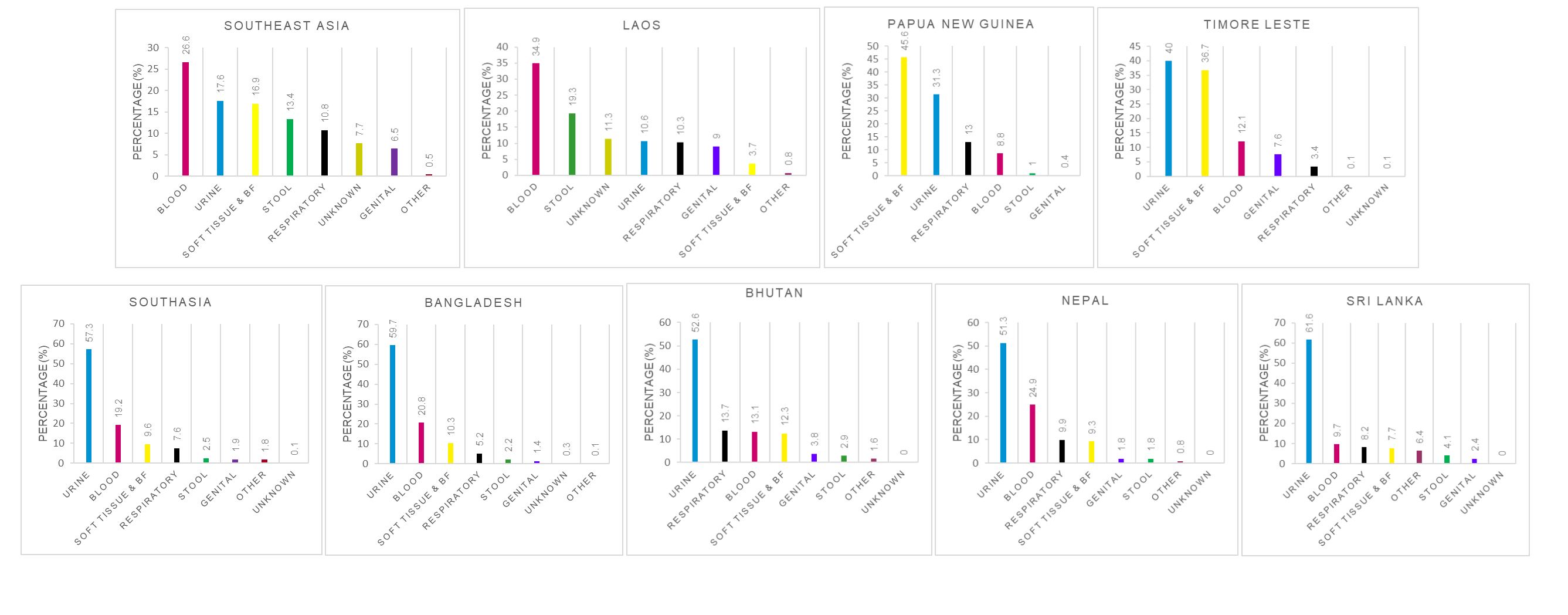

Supplement: ciad585_Supplementary_Data [file ciad585_supplementary_data.zip › Supplementary Figure 2.tiff]
